# Tumor-infiltrating mucosal-associated invariant T (MAIT) cells retain expression of cytotoxic effector molecules

**DOI:** 10.18632/oncotarget.26866

**Published:** 2019-04-19

**Authors:** Patrik Sundström, Louis Szeponik, Filip Ahlmanner, Malin Sundquist, Justin S.B. Wong, Elinor Bexe Lindskog, Bengt Gustafsson, Marianne Quiding-Järbrink

**Affiliations:** ^1^ Department of Microbiology and Immunology, Sahlgrenska Academy at University of Gothenburg, Göteborg, Sweden; ^2^ Department of Pathology, National University Hospital, Singapore and Department of Microbiology, National, University of Singapore, Singapore; ^3^ Department of Surgery, Sahlgrenska Academy at University of Gothenburg, Göteborg, Sweden

**Keywords:** tumor immunity, MAIT cells, cytotoxicity, colon adenocarcinoma, granzyme B

## Abstract

Mucosal-associated invariant T (MAIT) cells all express a semi-invariable T cell receptor recognizing microbial metabolites presented on the MHC class I-like molecule MR1. Upon activation, they rapidly secrete cytokines and increase their cytotoxic potential. We showed recently that MAIT cells with Th1 phenotype accumulate in human colon adenocarcinomas. Here, we investigated the cytotoxic potential of tumor-infiltrating MAIT cells in colon adenocarcinomas, and to what extent it may be affected by the tumor microenvironment. Activation of MAIT cells from tumors induced increased Granzyme B, and to a lesser extent, perforin expression. Degranulation was assessed by surface expression of CD107a, and was also seen in response to cognate antigen recognition. The cytotoxic potential of tumor-associated MAIT cells was very similar to that of MAIT cells from unaffected colon. MAIT cells were also identified by immunofluorescence in direct contact with tumor cells in sections from colon cancer specimens. To summarize, tumor-associated MAIT cells from colon tumors have strong cytotoxic potential and are not compromised in this regard compared to MAIT cells from the unaffected colon. We conclude that MAIT cells may contribute significantly to the protective immune response to tumors, both by secretion of Th1-associated cytokines and by direct killing of tumor cells.

## INTRODUCTION

Mucosal-associated invariant T (MAIT) cells are semi-invariant T cells expressing a T cell receptor (TCR) comprising Vα7.2 joined with Jα33, which in turn are combined with a limited selection of Vβ chains [1, 2]. The resulting TCR recognizes microbial metabolites of vitamin B2 (riboflavin) which are synthesized by many bacterial species, as well as fungi. These metabolites are presented by the highly conserved and invariant MHC-Ib major histocompatibility complex-related protein 1 (MR1) molecule, present on both classical antigen-presenting cells and different types of epithelial cells [3, 4]. Recent reports have also identified minor populations of Vα7.2^–^ MAIT cells, which are still MR1-restricted but react to other microbial products than riboflavin metabolites [5, 6].

MAIT cells are present in the circulation and peripheral organs like liver, lungs, and the gastrointestinal mucosa. Indeed, MAIT cells efficiently leave the circulation and enter inflamed tissues without the need for phenotypic changes or activation in lymphoid organs [7]. When activated by cognate antigen recognition or by combinations of cytokines, MAIT cells rapidly secrete cytokines and up-regulate cytotoxicity-related molecules like Granzyme B (GrB) [8–10]. Depending on context, MAIT cells secrete a combination of Th1- and Th17-related cytokines such as IFN-γ, TNFα, IL-17 and IL-22, and the ratio between the different cytokines vary with tissue localization and stimulus [11]. Of note, efficient MR1-mediated MAIT cell activation requires uptake of intact bacteria by the antigen-presenting cells [12]. However, antigen-independent activation of MAIT cells through microbe-induced secretion of cytokines such as IL-12 and IL-18 probably contributes substantially to immunity to both bacterial and viral infections [12–14]. The broad distribution of their cognate antigens, the ability of epithelial cells to rapidly activate MAIT cells [15, 16], and their location in mucosal tissues have led to suggestions that MAIT cells constitute a first line of defense against invading microorganisms. However, MAIT cells have also been implicated in immunopathology in autoimmune diseases [17, 18], inflammatory bowel disease [19, 20] and the immune dysregulation in adipose tissues of patients suffering from type 2 diabetes [21].

MAIT cells are present in both the lamina propria and intraepithelial compartment of the colon mucosa, and colon lamina propria MAIT cells have a Th1-dominated cytokine profile [22–24]. It was recently demonstrated that the majority of MAIT cells in the lungs and liver are tissue-resident memory T (T_RM_) cells [25]. Gastrointestinal MAIT cells express CD69 and CD103 [22, 26, 27] and a large proportion may thus also be T_RM_ cells. However, we did not investigate effector functions in relation to T_RM_ cells. It should be noted, though, that at least some human MAIT cells recirculate and can be detected in thoracic lymph [28].

We and others have recently shown an accumulation of MAIT cells in colon adenocarcinomas [22, 29, 30], and our previous study demonstrated a predominant Th1 profile of tumor-infiltrating MAIT cells with production of IFN-γ and TNFα. However, the production of IFN-γ from tumor-infiltrating MAIT cells was found to be significantly lower compared to that in the unaffected tissue. In addition to cytokines promoting anti-tumor immunity, the cytotoxic capacity of MAIT cells may be particularly relevant in the tumor microenvironment, as it may also contribute to anti-tumor immune defense. Circulating MAIT cells up-regulate cytotoxic molecules like GrB and perforin upon challenge with *E. coli*-loaded antigen-presenting cells, and are capable of degranulation and killing of bacterially infected cells [8–10]. Recent reports also suggest that tumor cells can be killed by circulating MAIT cells [29, 31]. However, reduced GrB production in MAIT cells isolated from liver metastases of colorectal cancer has recently been reported [32]. Still, there is little information about the cytotoxic capacity of MAIT cells from intestinal tissues or from tumors. Based on these considerations, we investigated the cytotoxic potential of MAIT cells from colon tumors and compared it to MAIT cells isolated from the unaffected colon and peripheral blood from the same patients. These studies are the first to provide a detailed assessment of the cytotoxic potential of tumor-infiltrating MAIT cells, and show that tumor-infiltrating MAIT cells have substantial cytotoxic capacity at the same level as cells isolated from unaffected tissue. Our results thus suggest that MAIT cells may contribute to local anti-tumor immunity by both secretion of Th1 cytokines and direct killing of tumor cells.

## RESULTS

### Cytotoxic potential of *ex vivo* mucosal MAIT cells

Cytotoxic T cells are one of the most important lymphocyte subsets correlating to immune-mediated protection against tumors [33–37]. To determine if tumor-associated MAIT cells may also contribute to anti-tumor cytotoxicity, we examined the cytotoxic potential of freshly isolated MAIT cells from colon tumors and unaffected colon tissue as well as peripheral blood from the same patients. MAIT cells were defined as CD45^+^CD3^+^ TCR γ/δ^–^CD4^–^Vα7.2^+^CD161^high^ cells, and the gating strategy is shown in Supplementary Figure 1A. In this patient material, MAIT cells constituted 0.3 to 37% of all CD8α^+^ T cells (median 3.3%) in the tumors, and this was significantly higher than in the unaffected tissue (median 2.1%; *p* < 0.001) but not compared to the blood (median 3.1%; Supplementary Figure 1B). This MAIT cell accumulation in tumors was also evident when comparing MAIT cell frequencies among all CD3^+^ T cells (Supplementary Figure 1C). There were no differences in MAIT cell frequencies in the tissues between men and women, or correlation with age in this middle aged to elderly population (Supplementary Figure 2). The former finding is in contrast to our previous study [22] were men were found to harbor more MAIT cells in unaffected colon tissue than women. However, with the larger number of patients now available for analysis, there is no significant difference between sexes with regard to MAIT cell frequencies. Furthermore, TNM stage and microsatellite status did not affect frequencies of tumor-infiltrating MAIT cells, even though there was a non-significant tendency of lower MAIT cell frequencies in more advanced tumors (Supplementary Figure 2). These findings confirm our previous observation of MAIT cell accumulation in colon tumors in an independent patient sample [22].

**Figure 1:**
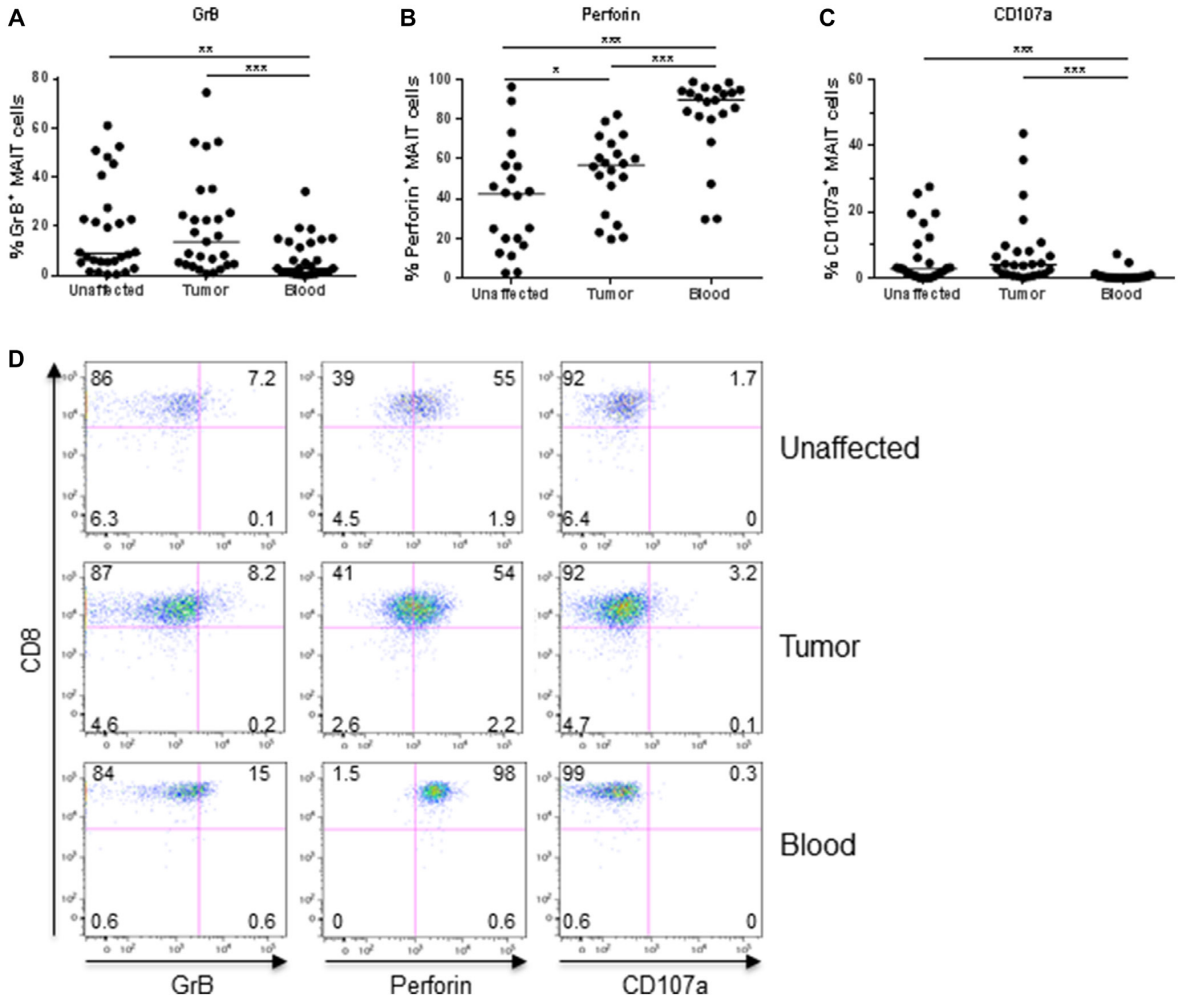
Frequencies of GrB^+^, Perforin^+^, and CD107a^+^ MAIT cells *ex vivo* Single cell suspensions were isolated from unaffected colon, colon tumors and peripheral blood, and MAIT cells analyzed for their expression of (**A**) GrB, (**B**) Perforin, and (**C**) CD107a by flow cytometry. (**D**) Representative dot-plots from one patient. Symbols represent individual values and the line the median. ^*^p < 0.05, ^**^p < 0.01, ^***^p < 0.001, *n* = 20–28.

**Figure 2:**
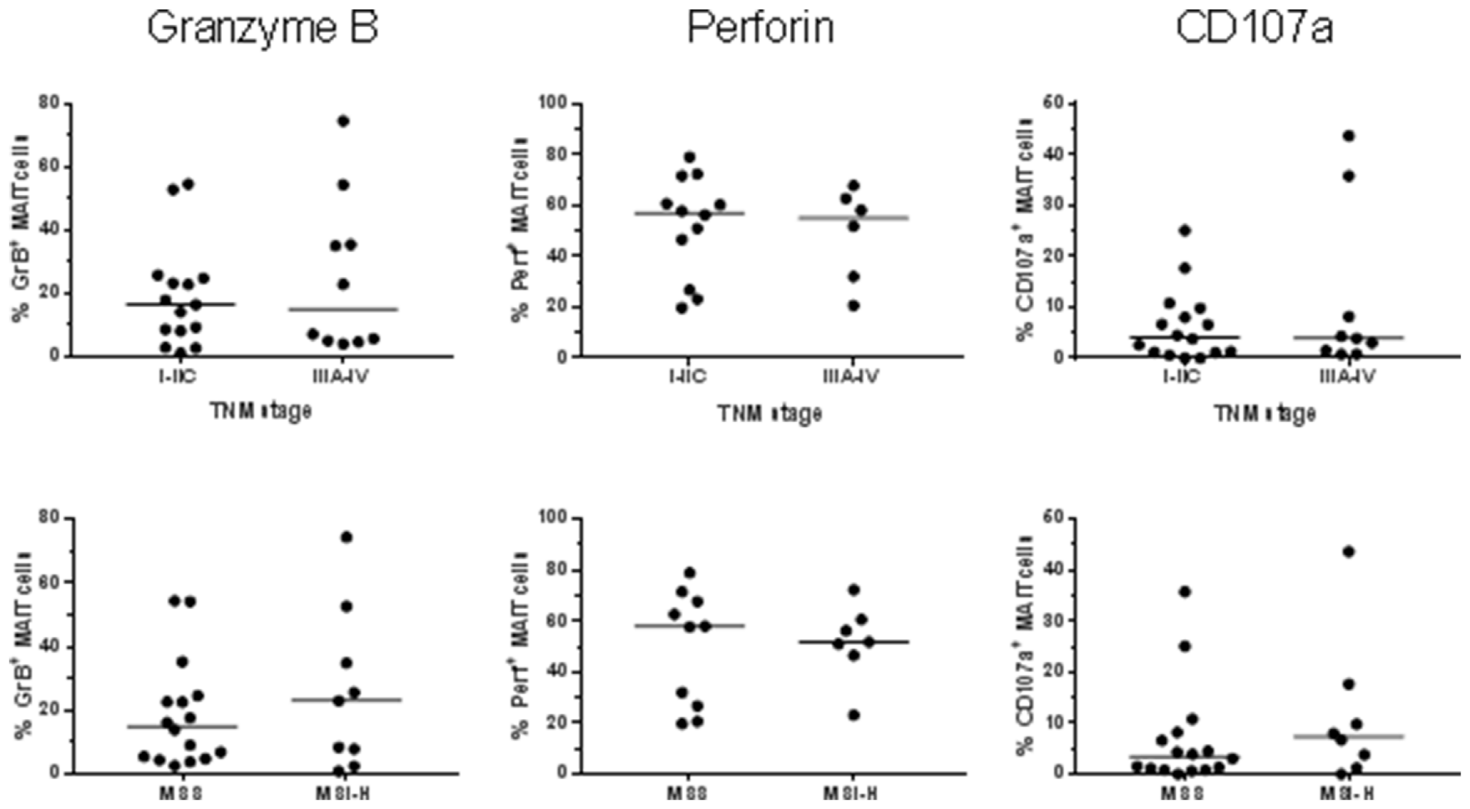
MAIT cell expression of cytotoxic molecules in relation to tumor stage and microsatellite instability Single cell suspensions were prepared from colon tumors, and the MAIT cell expression of GrB, Perforin and CD107a was determined by flow cytometry in freshly isolated cells. TNM stage and microsatellite status were retrieved from the pathology report. *n* = 17–25.

*Ex vivo* analyses showed that the expression of GrB in MAIT cells from colon tissues varied considerably between individuals. However, in both the unaffected tissue and tumors, GrB expression was significantly higher than in circulating MAIT cells (*p* < 0.01; Figure 1A, 1D). As we have previously shown in a smaller patient sample, there was no significant difference in the GrB expression between MAIT cells from tumors and unaffected tissue. Perforin expression, on the other hand, was significantly higher in MAIT cells from the tumors compared to the unaffected tissue (*p* < 0.05), but also here, expression varied substantially between individuals. Furthermore, circulating MAIT cells showed an even higher expression of perforin than colon MAIT cells (*p* < 0.001; Figure 1B, 1D). Surface expression of CD107a, a marker of recent degranulation was low in all the MAIT cell populations examined, but still significantly higher in the colon-resident and tumor-infiltrating MAIT cells compared to circulating (*p* < 0.001; Figure 1C, 1D). Furthermore, GrB expression in MAIT cells correlated positively between tumor and unaffected tissue from the same patient (*p* < 0.001, *R*^2^ = 0.377) as did perforin (*p* < 0.01, *R*^2^ = 0.428), but not CD107a expression. On the other hand, there was no correlation between the different molecules in the tumor-associated MAIT cells. In this somewhat limited material, there was no significant correlation between the *ex vivo* expression of the examined cytotoxic effector molecules by tumor-infiltrating MAIT cells and tumor TNM stage or microsatellite status (Figure 2).

In summary, these experiments show that tumor-associated MAIT cells express markers of cytotoxicity to the same or a higher extent than MAIT cells in the unaffected colon when analyzed *ex vivo*. Furthermore, colonic MAIT cell expression of cytotoxicity-related proteins was markedly different from that in circulating MAIT cells, with higher GrB and CD107a expression, and lower perforin expression.

### Induction of cytotoxic effector molecules in MAIT cells from different locations

**Figure 3:**
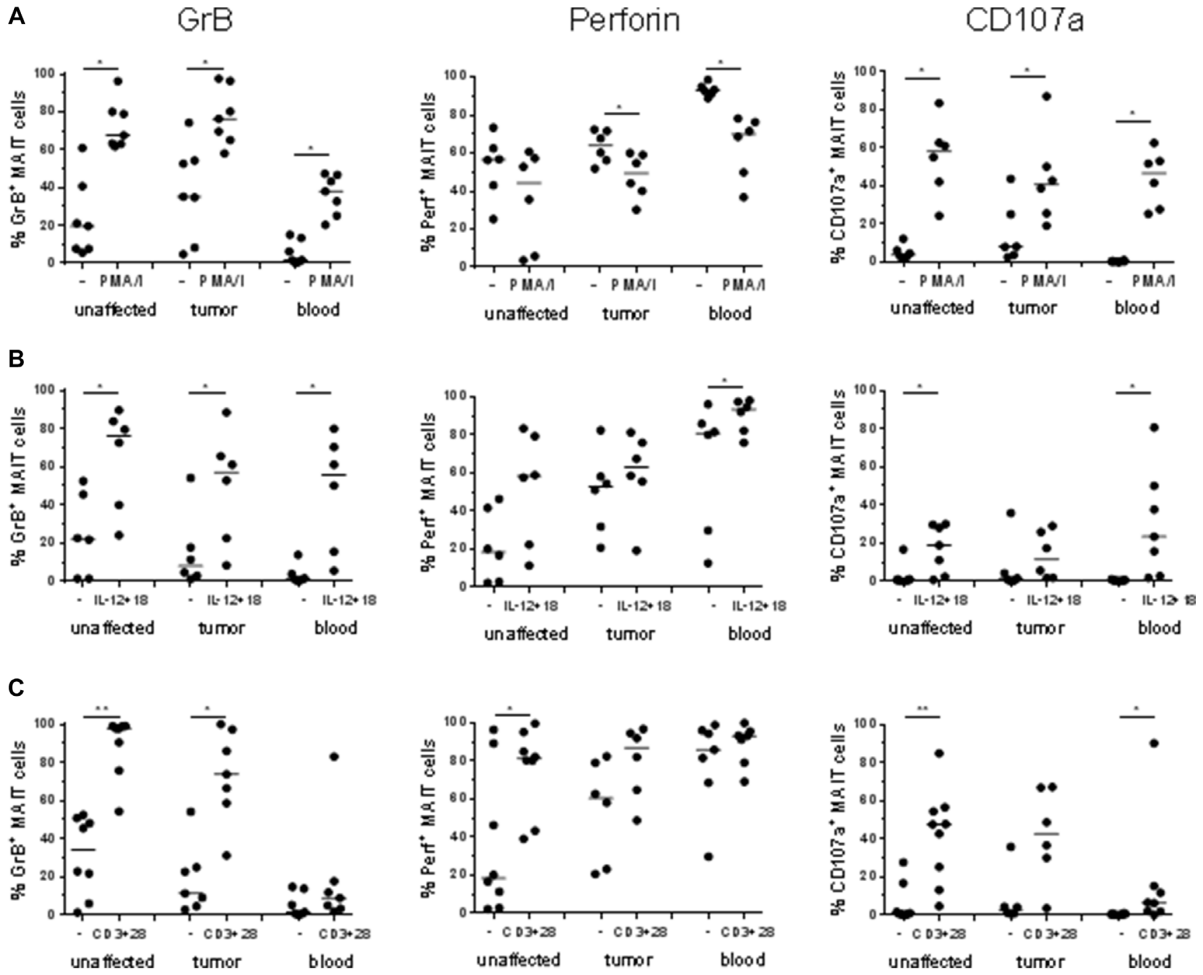
Frequencies of GrB^+^, Perforin^+^ and CD107a^+^ MAIT cells after stimulation Single cell suspensions were isolated from unaffected colon, colon tumors and peripheral blood, and stimulated with (**A**) PMA and Ionomycin, (**B**) IL-12 and IL-18, or (**C**) anti-CD3 and anti-CD28. Expression of GrB, Perforin, and CD107a was examined by flow cytometry. Symbols represent individual values and the line the median. *n* = 6–9 ^*^p < 0.05, ^**^p < 0.01.

We and others have previously shown that polyclonal MAIT cell activation will increase the expression of cytotoxic molecules in circulating and intestinal MAIT cells [8, 9, 22, 29, 32]. Here, however, we decided to examine several different modes of stimulation and assessed GrB, perforin, and CD107a expression to get a more complete assessment of the cytotoxic potential of tumor-associated MAIT cells. Following polyclonal activation of MAIT cells with PMA and Ionomycin, there was a consistent up-regulation of GrB in MAIT cells from both the tumors and the unaffected tissue, as well as from blood (Figure 3A, Supplementary Figure 3). As shown in Figure 1A, GrB expression varies considerably in unstimulated MAIT cells, but in all individuals, we could detect a robust increase in MAIT cell GrB expression after stimulation, both in tumors and unaffected tissues (*p* < 0.05). In contrast, perforin expression was not increased by polyclonal stimulation with PMA and Ionomycin, but instead decreased following stimulation.

The combination of IL-12 and IL-18 has previously been shown to induce potent cytokine responses in human MAIT cells [13], and this cytokine combination also induced up-regulation of GrB in MAIT cells from all examined tissues (*p* < 0.05), as well as a further increase in perforin expression in the circulating MAIT cells (*p* < 0.05; Figure 3B).

In addition, TCR-mediated stimulation with anti-CD3 and anti-CD28 antibodies also induced a robust GrB response in colon MAIT cells from both tumors and unaffected tissues (*p* < 0.05; Figure 3C). In stark contrast, circulating MAIT cells from the same individuals were mostly unresponsive to TCR-mediated stimulation, again highlighting the differences between circulating and colon-resident MAIT cells. Perforin expression was significantly increased in MAIT cells from unaffected colon following TCR-mediated stimulation (*p* < 0.05) but not in tumor-infiltrating or circulating MAIT cells (Figure 3C).

When circulating MAIT cells from healthy volunteers were stimulated the same ways, the responses were essentially similar to those seen in blood-derived MAIT cells from cancer patients, but generally of a lower magnitude (Supplementary Figure 4). This observation reinforces the view that MAIT cells from colon cancer patients are not deficient in their cytotoxic responses. Taken together, MAIT cells from both tumors and unaffected colon tissue potently up-regulate cytotoxic granule content in response to different types of polyclonal stimulation. In addition, there were no major differences between the two subsets in their ability to respond to any of the different stimulations.

### Degranulation of tumor-associated MAIT cells following bacterial stimulation

Degranulation is a pre-requisite for GrB- and peforin-mediated killing of target cells by cytotoxic T cells. Surface expression of the lysosomal protein CD107a (LAMP1) is commonly used to detect recent degranulation in T cells [38]. As seen in Figure 1C, CD107a expression is low in MAIT cells from both blood and colon tissue *ex vivo*. Polyclonal activation with PMA and Ionomycin led to a substantial degranulation of MAIT cells from all three examined tissues (*p* < 0.05, Figure 3A, Supplementary Figure 5), while stimulation with cytokines was less efficient in increasing CD107a surface expression (Figure 3B). TCR-mediated stimulation, on the other hand, increased surface expression of CD107a (*p* < 0.05, Figure 3C), but as with GrB expression, the effect was usually more prominent in colon-derived than circulating MAIT cells.

**Figure 4:**
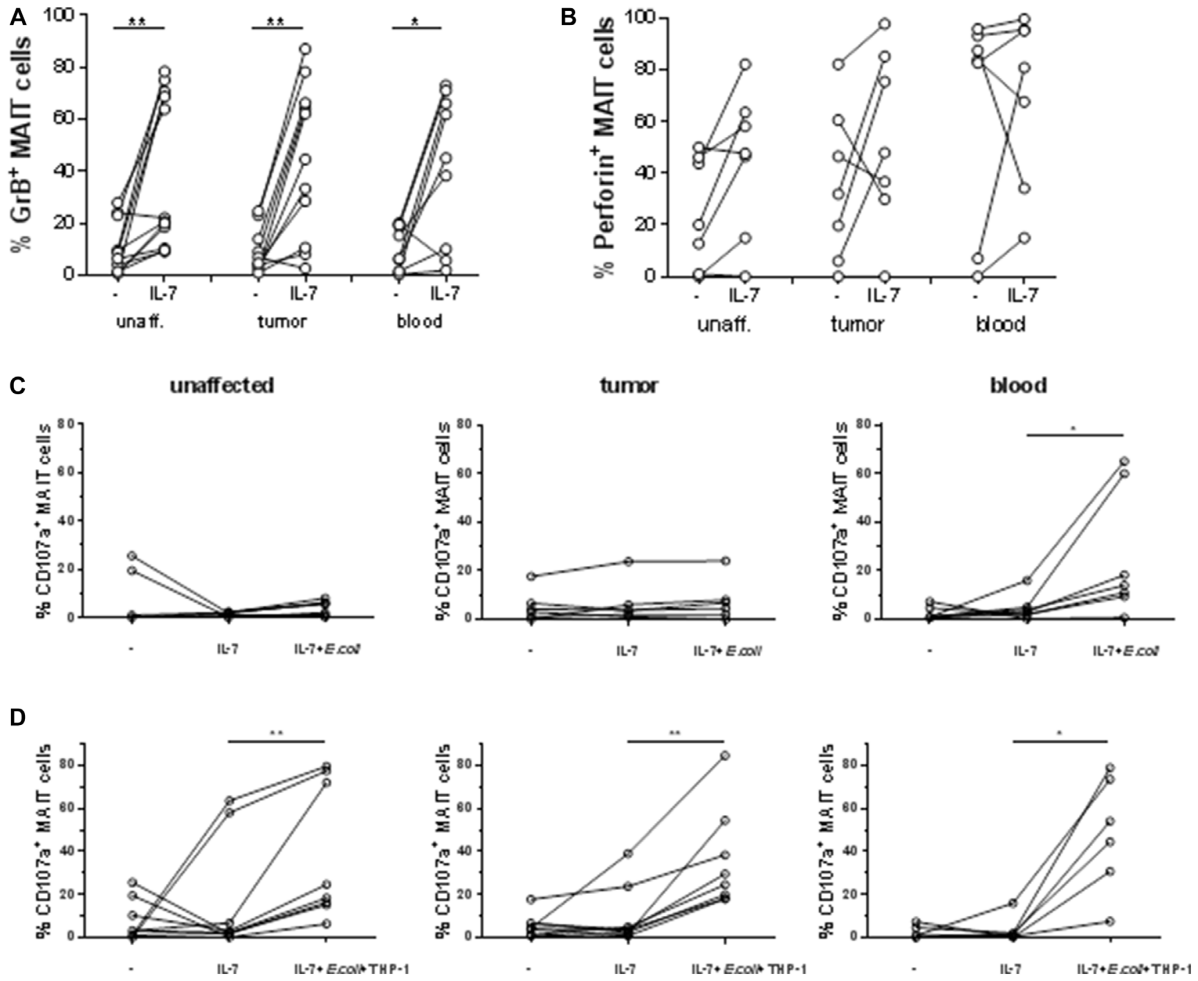
Priming and degranulation of intestinal MAIT Single cell suspensions were isolated from unaffected colon, colon tumors and peripheral blood, and incubated alone or with IL-7 for 48 hours. Expression of GrB (**A**) and perforin (**B**) was examined by flow cytometry. They were then either left in IL-7 or stimulated with (**C**) fixed *E. coli* bacteria or (**D**) THP-1 cells pre-incubated with fixed *E. coli* for 4 hours, both in the presence of IL-7. Expression of CD107a was examined by flow cytometry. Symbols represent individual values and lines the median. *n* = 6–12 ^*^p < 0.05, ^**^p < 0.01.

Recent studies have demonstrated that IL-7 primes MAIT cells from the circulation for cytotoxic activity [39], and here we investigated if MAIT cells from colon tissue and tumors respond similarly to IL-7. Culture of freshly isolated MAIT cells with IL-7 induced increased expression of GrB in MAIT cells from all three tissues examined in most of the individuals (*p* < 0.05) while there was no significant change in Perforin expression after incubation with IL-7 (Figure 4A–4B). However, IL-7 alone did not induce degranulation, as measured by CD107a expression (Figure 4C). It has been demonstrated by Leeansyah et al [10] that MAIT cells do not de-granulate to any larger extent unless they get triggered with specific antigen. We thus added formalin-fixed *E. coli* to the cell suspensions cultured with IL-7 and measured cytotoxic capacity and degranulation in cell suspensions from tissues and blood. Circulating MAIT cells started displaying CD107a on their surface following antigenic stimulation with *E. coli* (*p* < 0.05; Figure 4C). In stark contrast, MAIT cells from tumors and unaffected colon did not increase expression of CD107a at all. Reasoning that this may be due to a shortage of MR-1 expressing cells with ability to process and present the bacterial antigen to MAIT cells in the colon-derived cell suspensions, e.g. epithelial cells and dendritic cells which are not present to any larger degree in the lamina propria cell suspensions, we pre-incubated the myelomonocytic cell line THP-1 with fixed *E. coli* and used these cells as antigen-presenting cells. In this setting, inclusion of efficient antigen-presenting cells led to a substantial and consistent amount of CD107a on the surface also of colon-derived MAIT cells (*p* < 0.01; Figure 4D). In some individuals, GrB expression in MAIT cells was further increased upon co-incubation with antigen-loaded THP-1 cells, but usually, the pre-incubation with IL-7 alone was sufficient to induce GrB (Supplementary Figure 6). Degranulation was reduced by >30% when blocking antibodies to MR1 were added to the tumor-derived cultures, showing that cognate antigen recognition partly contributes to MAIT cell degranulation (Supplementary Figure 7). To formally rule out bystander effects of other cell types present in tissue-derived cell suspensions on MAIT cells, we isolated Vα7.2^+^ T cells from unaffected colon and tumors, and co-cultured them with antigen-presenting THP-1 cells. Similarly to what we found in unfractionated preparations of tumor-infiltrating lymphocytes, co-culture with antigen-presenting THP-1 induced a moderate increase in GrB expression in the Vα7.2^+^CD161^high^ MAIT cells, as well as substantially increased surface expression of CD107a. In contrast, Vα7.2^+^CD161^low^ non-MAIT cells also present in the MAIT cell suspension did not change their expression of GrB or CD107a (Supplementary Figure 8). Again, there was no consistent difference in the degranulation between MAIT cells isolated from the unaffected tissue and the tumors.

In conclusion, these results show that IL-7 primes intestinal MAIT cells for cytotoxicity, and that antigen-specific stimulation leads to release of cytotoxic granules to the same extent in colonic and tumor-associated MAIT cells.

### Localization of MAIT cells in colon tumors

**Figure 5:**
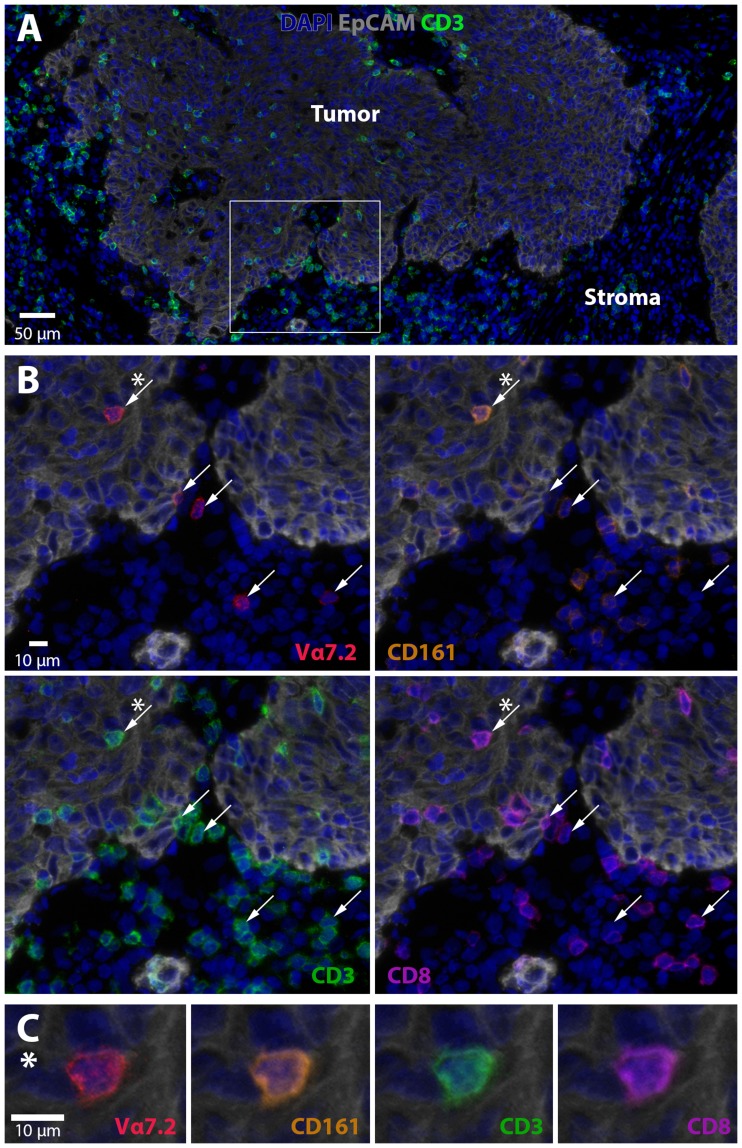
MAIT cell localization in colon tumors Frozen section of a tumor located in the ceakum was stained with DAPI to visualize nuclei (blue), EpCAM (grey) for epithelial cells, CD3 (green), CD8 (purple), CD161 (orange), and Vα7.2 (red). (**A**) Overview of the tumor (EpCAM^+^) and tumor stroma (EpCAM^–^) together with nuclei and CD3 staining. A magnified area in (**B**) shows Vα7.2, CD161, CD8, and CD3. Vα7.2^+^ cells are indicated with arrows. The cell marked with a star (^*^) is shown in (**C**) and demonstrates co-localization of CD3, CD8, CD161, and Vα7.2 on a tumor-infiltrating T cell. One representative experiment out of three is shown.

There is no single marker that can be used to identify MAIT cells in tissue sections, as some conventional T cells also express Vα7.2 in combination with other β-chains. We thus established a multi-color immunofluorescence panel to detect CD3^+^CD8^+^Vα7.2^+^CD161^+^ putative MAIT cells in frozen tissue sections and combined these markers with EpCAM to identify the tumor cells. These analyses showed Vα7.2^+^CD161^+^ T cells in both the tumor tissue itself and in the tumor stroma (Figure 5). These putative MAIT cells were always CD3^+^, but both CD8^+^ and CD8^–^. Still, the majority (68 ± 14%) were CD8^+^. Quantification of in this limited material showed that 18.2 ± 15.0% (mean ± SD, *n* = 3) of CD8^+^ cells in the stroma were putative MAIT cells and 6.2 ± 3.9 of the CD8^+^ cells in direct contact with tumor cells. When considering MAIT cell density, the stroma contained 50 ± 29 putative MAIT cells/mm^2^ and the tumor epithelium 12 ± 8. These findings strongly suggest that a fraction of MAIT cells are in a physical location that makes it possible for them to recognize antigens presented by tumor cells and release cytotoxic granules. Thus, MAIT cells with cytotoxic potential may contribute significantly to anti-tumor immunity by killing tumor cells.

## DISCUSSION

MAIT cells accumulate in colon tumors, and produce Th1-related cytokines [22]. However, the cytotoxic capacity of tumor-associated MAIT cells has not been properly assessed to date. In this study, we show that tumor-infiltrating MAIT cells express cytotoxic effector molecules and degranulate in response to antigen stimulation. They are also localized in close proximity to tumor cells in patient tissue samples. Furthermore, there is no functional impairment of MAIT cells in tumors with regard to cytotoxic abilities, when comparing to MAIT cells in unaffected colon mucosa.

Several studies have shown a positive correlation between colon tumor infiltration of conventional cytotoxic CD8^+^ T cells and patient outcome [33–37]. As many of the organisms in the normal microbiota of the large intestine can provide the antigens recognized by MAIT cells [15, 40], and since the epithelial barrier is poor in colon tumors [41], MAIT cell activation by antigens derived from the microbiota and subsequent cytokine production and cytotoxic activity may be important for the outcome of colorectal cancer. However, our previous studies demonstrated that tumor-resident MAIT cells have a reduced ability to secrete IFN-γ upon stimulation, and also indicated that factors in the tumor microenvironment may render MAIT cells unresponsive [22]. A similar and even more pronounced reduction in cytokine secretion was recently described in liver metastases of colorectal cancer, further implicating tumor microenvironmental factors in the function of MAIT cells [32]. Based on these considerations, we determined the production of cytotoxic effector molecules by tumor-associated MAIT cells, in an attempt to investigate if their cytotoxic activity may contribute to anti-tumor immunity. These studies demonstrated that there was no impairment in cytotoxic ability in MAIT cells isolated from tumors compared to the unaffected tissue. If anything, tumor-infiltrating MAIT cells had a higher expression of cytotoxicity markers *ex vivo* compared to MAIT cells from unaffected tissues. This may well be a result of the aforementioned barrier defect reported in colon tumors [41]. In addition, MR1 is expressed in colon tumor tissue [22] and there would thus be ample possibilities for local presentation of microbial antigens to tumor-infiltrating MAIT cells.

Another interesting observation from the *ex vivo* studies is the difference in expression of cytotoxic proteins between circulating and tissue-localized MAIT cells. Circulating MAIT cells expressed less GrB and CD107a than tissue-resident MAIT cells, and this is not entirely surprising, as intestinal MAIT cells would be more likely to encounter MR1^+^ cells presenting bacterial antigens. The finding that circulating MAIT cells expressed higher levels of perforin than colonic MAIT cells was more unexpected. Still, these results all emphasize the importance of studying MAIT cells from relevant tissue samples rather than approximating a function from circulating MAIT cells, even if collected from patients. This notion is further underscored by the different responses that we recorded in tissue and circulating MAIT cells following TCR-mediated stimulation.

As previously demonstrated [22, 42], polyclonal activation of both circulating and colon-derived MAIT cells with PMA and Ionomycin induced robust GrB responses, also in the patients with high *ex vivo* GrB expression. The same was seen for cytokine-mediated stimulation with IL-12 and IL-18. Cytokine-mediated induction of GrB has previously been shown with circulating MAIT cells [10, 43], and we now extend these findings to intestinal and tumor-derived MAIT cells. Priming of MAIT cells for cytotoxic activity can also be achieved by IL-7 [10, 18], and here we show similar responses also to IL-7 in circulating and intestinal MAIT cells. The increased cytotoxicity in tumor-infiltrating MAIT cells in response to cytokines is interesting, as it indicates possible new ways to improve anti-tumor immunity by manipulating MAIT cells, potentially by stimulating stromal cells to secrete cytokines by triggering innate pattern recognition receptors.

In contrast to cytokine-mediated activation of MAIT cells, stimulation with antibodies to CD3 and CD28 yielded very different outcomes in colon-derived and circulating MAIT cells, as circulating MAIT cells responded poorly to TCR-mediated stimulation, with regard to both upregulation of cytotoxic molecules and degranulation. Two recent studies have convincingly demonstrated that full TCR-mediated MAIT cell activation is dependent both on priming by TLR ligands or pro-inflammatory cytokines and on antigen recognition [43, 44]. These studies focused mainly on cytokine responses, but our current results indicate that the same conclusions are probably valid also for cytotoxic responses. The differential responsiveness between intestinal and blood MAIT cells may thus be another reflection of a need for a pro-inflammatory signal delivered by cytokines or microbial products before full responsiveness to TCR-mediated stimulation can be achieved. In the colon, this signal would be provided by the organisms in the normal microbiota, which would not be available for MAIT cells residing in the circulation. The low, but still detectable, expression of CD107a on MAIT cells from colon tissues would also suggest a certain degree of granulae release already *in vivo*. It should be noted, though, that another study recorded increased GrB expression in circulating MAIT cells following TCR-mediated stimulation in combination with antibodies to CD2 [8]. The regulation of perforin expression was similar to that of GrB, with the exception of PMA/Ionomycin stimulation, which actually led to decreased perforin expression in cells from both blood and tissue. While stimulation with *E. coli*-derived antigen up-regulates perforin expression [8, 45], one study shows an unchanged, very low expression of perforin following PMA/Ionomycin stimulation [29]. Differences may depend on different stimulation times and antibody clones, but clearly this issue needs more attention in future studies.

We then turned to assess the degranulation potential of tumor-infiltrating MAIT cells. IL-7 alone increased the expression of GrB and Perforin to the same extent in tumor-infiltrating MAIT cells and their counterparts in the unaffected tissue, but did not lead to degranulation, as measured by CD107a. However, when IL-7-primed MAIT cells from the circulation were exposed to bacterial antigens, they readily responded by granule release, as previously reported [8–10]. When extending these studies to intestinal and tumor-infiltrating MAIT cells, we detected prominent degranulation in cells from all the patients after antigen-specific stimulation. We also found that intestinal MAIT cells were dependent on external addition of antigen-presenting cells to respond to *E. coli*-derived antigens. This observation does not preclude *in vivo* activation of MAIT cells in colon tissues, as important antigen presenting cells such as dendritic cells and macrophages [46] are not isolated optimally with the current protocol, which is developed for isolation of lymphocytes. In addition, epithelial cells are removed during the isolation procedure. Of note, there were no differences in the degranulation of MAIT cells from unaffected and tumor tissues. A recent study showed that MAIT cells from the gastric mucosa could respond to *Helicobacter pylori*-infected THP-1 cells with increased CD107a expression [26]. However, gastric MAIT cells displayed considerably less de-granulation than colon- or tumor-derived MAIT cells in the current study, but this may be due to their physical location, the different bacteria used to trigger degranulation, or the lack of IL-7 priming of the gastric MAIT cells. We conclude that intestinal MAIT cells mount potent cytotoxic responses to all tested polyclonal stimuli and to antigen-specific stimulation, possibly indicating priming *in vivo* by relevant microbial co-stimulatory signals. In addition, we find no impairment in the cytotoxic potential or response by tumor-infiltrating MAIT cells. Thus, the putative suppressive factors present in colon tumors seem to affect Th1 cytokine production rather than cytotoxicity of MAIT cells. Taken together, our findings indicate that tumor-infiltrating MAIT cells may complement conventional CD8^+^ cytotoxic T cells, and may provide a mechanism for killing of tumor cells, even in the absence of tumor cell-derived peptide antigens.

To further investigate to which extent MAIT cells may actually be able to kill tumor cells, we also determined their localization in the tissue microenvironment. Tissue sections demonstrate that MAIT cells can be found in close contact with tumor cells, both in the transformed epithelium and directly beneath. It has previously been shown that MAIT cells can kill epithelial cells infected with bacteria [15, 16], and we propose that transformed colon epithelial cells may also be killed by MAIT cells if presenting bacterial antigens. MAIT cell density in tumors *per se* is not correlated to patient outcome, while a relative accumulation compared to the unaffected tissue correlated to a less favorable clinical outcome [30]. We have not been able to follow the patients in this study long enough to determine outcome in relation to cytotoxic markers. However, we note that MAIT cell density or expression of cytotoxic molecules *ex vivo* do not correlate to tumor stage or microsatellite status in our patient material. The fact that MAIT cells are more resistant to chemotherapy than conventional T cells [42] may make them important for retaining anti-tumor immune responses during treatment.

Taken together, our data demonstrate that the MAIT cells infiltrating colon tumors have cytotoxic potential, and that they are not compromised in this regard compared to MAIT cells from the unaffected colon. We conclude that MAIT cells may make an important contribution to the protective immune response to colon tumors, both by secretion of Th1-associated cytokines and by direct killing of tumor cells. The possibility to boost tumor-infiltrating MAIT cell cytotoxic functions by cytokines may open up for new therapeutic options to further improve anti-tumor immunity.

## MATERIALS AND METHODS

### Patients and tissue collection

This study was performed according to the Declaration of Helsinki and approved by the Regional Board of Ethics in Medical Research in west Sweden. All volunteers gave a written informed consent before participation. Altogether, 35 individuals undergoing curative resection of colon tumors at the Sahlgrenska University Hospital were included in the studies 16 males and 19 females, aged 37 to 93, median age 75). Additional patient data is presented in Supplementary Table 1. None of the patients suffered from autoimmune disease, were on immunomodulatory drugs, or had undergone radiotherapy or chemotherapy for at least three years prior to colectomy. Immediately after colectomy, a section of the tumor tissue encompassing both the center and more peripheral parts of the tumor was collected, as well as unaffected tissue from at least ten centimeters away from the tumor. Biopsies from the resection material were placed in OCT medium and immediately frozen in iso-pentane, followed by liquid nitrogen, and subsequently stored at –80°C until analysis. The remaining colectomy material was transported in ice-cold PBS before isolation of lymphocytes within less than two hours. Heparinized venous blood was also obtained during surgery. Information about tumor stage, describing tumor invasion (T1-T4), lymph node involvement (N0-N2), and distant metastases (M0-M1) was retrieved from the pathology report. The TNM information is then combined to an overall stage (stage I-IV). Microsatellite instability (MSI), indicating the mutational load of the tumor, was analysed using the MSI Analysis System, Version 1.2 (ProMega) which includes fluorescently labeled primers for co-amplification of seven markers including five mononucleotide repeat markers (BAT-25, BAT-26, NR-21, NR-24 and MONO-27) and two pentanucleotide repeat markers (Penta C and Penta D). MSI was defined as peak alterations in the marker electropherogram in the tumor compared with corresponding normal tissue. A tumor was defined as MSI high (MSI-H) if > 1 of the 5 markers showed instability, and if no MSI was detected, the tumor was designated microsatellite stable (MSS).

Peripheral blood was also collected from 6 healthy volunteers (3 males and 3 females, aged 54 to 74, median 61) and used for lymphocyte isolation.

### Cell isolation and stimulation

Lamina propria lymphocytes were isolated essentially as described [22]. Briefly, the tissue samples were washed with PBS and the muscle layers, fat, connective tissue and blood vessels were carefully removed. The tissue was cut into 5 mm pieces and subjected to four rounds of EDTA treatment to remove epithelial cells and intraepithelial lymphocytes. The remaining tissue was digested with Liberase TM (Roche) together with DNase I (Sigma Aldrich) for 2 hours. The resulting single cell solution was re-suspended in RPMI 1640 (GIBCO^®^ by Life Technologies^™^) containing 10% fetal bovine serum (Biological Industries), 25 mM of hepes, 100 U/ml of penicillin, 100 μg/ml of streptomycin, 292 μg/ml of L-glutamine (GIBCO™ Invitrogen Corporation), and 50 μg/ml of gentamicin (Lonza). The enzymes employed for lymphocyte isolation did not affect detection of the markers used for MAIT cell identification, as parallel enzymatic treatment of PBMC did not reduce the surface expression of CD161 or Vα7.2. PBMC were isolated by gradient centrifugation on Ficoll-Paque™Plus (GE Healthcare Bio-sciences AB).

To assess production of GrB and perforin, cells were stimulated with 50 ng/mL of PMA and 500 ng/mL of ionomycin calcium salt (Sigma Aldrich) for 12 hours, 50 ng/mL of IL-12 and 50 ng/mL of IL-18 (R&D Systems) for 40 hours [13], or bead-bound antibodies to CD3 and CD28 (Dynal AS) for 40 hours. For detection of cytokines, a protein transport inhibitor (BD Golgi stop, BD Biosciences) was added 4–12 hours before harvest of stimulated cells. For assessment of degranulation, single cell suspensions were rested over night at 37°C and then primed with 25 ng/ml of IL-7 (R&D SYSTEMS^®^) for 40 hours.

### Flow cytometry

Single cell suspensions were stained with CD4-FITC (clone OKT-4), TCR γ/δ-FITC (clone B1), TCR Vα7.2-APC (clone 3C10), CD107a-Brilliant Violet 650^™^ (clone H4A3), Perforin-PE (clone dG9) (All from Biolegend Inc., San Diego, USA), CD3-Brilliant Violet 711 (clone UCHT1), CD3-APC-H7 (clone SK7), CD8-Brilliant Violet 711 and -Alexa Fluor^®^ 700 (clone RPA-T8), CD45-PerCP (clone 2D1), GrB-PE and -Alexa Fluor^®^ 700 (clone GB11) (BD Biosciences™), and CD161-eFluor450 (clone HP-3G10) (eBioscience). Lymphocytes were identified by their forward and side scatter characteristics, and LIVE/DEAD Fixable Aqua Dead Cell Stain Kit (molecular probes^®^ by Life Technologies^™^) was used to gate out dead cells. FIX&PERM^®^ (AN DER GRUB Bio Research GmbH) intracellular staining kit was used for detection of cytokines. Isotype controls were used to determine cut-off levels for positive perforin staining, and cut-off levels for GrB and CD107a were guided by expression in circulating conventional CD8^+^ T cells, which have distinct GrB^+^ and GrB^–^ populations and are CD107a^–^. Data was acquired using a Becton Dickinson LSR II flow cytometer and analyzed by FlowJo software.

### Degranulation in response to antigen

Degranulation of IL-7 primed MAIT cells was analyzed essentially as described by Dias et al [39]. In some experiments, CD8^+^ T cells were first positively selected by magnetic beads (Dynal AS), followed by removal of the beads with Detachabead (Dynal) and a second round of positive selection of Vα7.2^+^ cells using APC-labelled anti-Vα7.2 antibodies and EasySep APC Selection Kit (STEMCELL^™^ Technologies). The resulting cell suspensions contained on average 80% Vα7.2^+^ T cells, and consisted of both CD161^high^ MAIT cells and CD161^neg/low^ conventional CD8^+^ T cells using Vα7.2 in their TCR.

THP-1 cells were generously donated by Dr Mats Bemark at the University of Gothenburg and grown in RPMI 1640 (GIBCO^®^ by Life Technologies) containing 10% fetal bovine serum (Biological Industries), 100 U/ml of penicillin, 100 μg/ml of streptomycin, 292 μg/ml of L-glutamine, 50 μg/ml of gentamicin (all from GIBCO), and 50 μg/ml of 2-mercaptoethanol (Lonza). To assess antigen-specific stimulation of MAIT cells, THP-1 cells were incubated for 4 hours with *E. coli* strain K12-C600 fixed for 20 minutes in 1% of formaldehyde at a cell:bacteria ratio of 1:5, washed and used as target cells. THP-1 cells and MAIT cells were co-cultured for 18 hours in the presence of antibodies to CD107a. After harvest, cells were stained for additional surface markers, fixed, and stained for GrB as described above.

### Immunofluorescence

OCT embedded tissue was cut in 7 μm thick sections and stored at –20°C. Sections were directly fixed in 50% ice-cold acetone for 30 sec followed by 100% acetone for 10 min, and air dried and washed in PBS. Following Avidin/Biotin blocking, CD161 was stained with anti-CD161 (clone HP-3G10, BD) followed by donkey anti-mouse-Cy3 (Fab_2_, Jackson Immuno). Subsequently sections were incubated with 5% mouse serum for 10 min, rinsed with PBS and then incubated with anti-CD3-BB515 (UCHT1, BD), anti-CD8-PerCP (RPA-T8, Biolegend), anti-EpCAM-594 (G8.8, Biolegend) and anti-Vα7.2-APC (3C10, Biolegend) in PBS with 0,1% FBS. Sections were incubated with anti-APC-biotin (APC003, Biolegend) followed by Streptavidin-BV480. The tissue was stained with DAPI and mounted in ProLong Antifade Diamond (Invitrogen). The primary antibody incubations were performed for 60 min at RT and secondary and tertiary incubations for 40 min at RT. Tissue sections were scanned with the Metafer Slide Scanning Platform (Axio Imager.Z2 Microscope and 20x/0.8/air objective, Zeiss).

### Statistical analyses

Statistical analyses were performed using two-tailed Wilcoxon matched-pairs signed rank test. Values of *p* < 0.05 were considered to be statistically significant.

## SUPPLEMENTARY MATERIALS FIGURES AND TABLE



## Abbreviations

GrB: Granzyme B
MAIT cell: mucosal-associated invariant T cell
MR1: major histocompatibility complex-related protein 1
MSI: microsatellite instability
MSS: microsatellite stable
TCR: T cell receptor
T_RM_ cell: tissue resident memory T cell
