# Supplementary material for: Tumor-infiltrating mucosal-associated invariant T (MAIT) cells retain expression of cytotoxic effector molecules
Source: Oncotarget. 2019 Apr 19;10(29):2810–23. doi: 10.18632/oncotarget.26866 (PMC6497460; doi:10.18632/oncotarget.26866)
Supplement: Supplementary file 1 [file oncotarget-10-2810-s001.pdf]

# Tumor-infiltrating mucosal-associated invariant T (MAIT) cells retain expression of cytotoxic effector molecules

## SUPPLEMENTARY MATERIALS

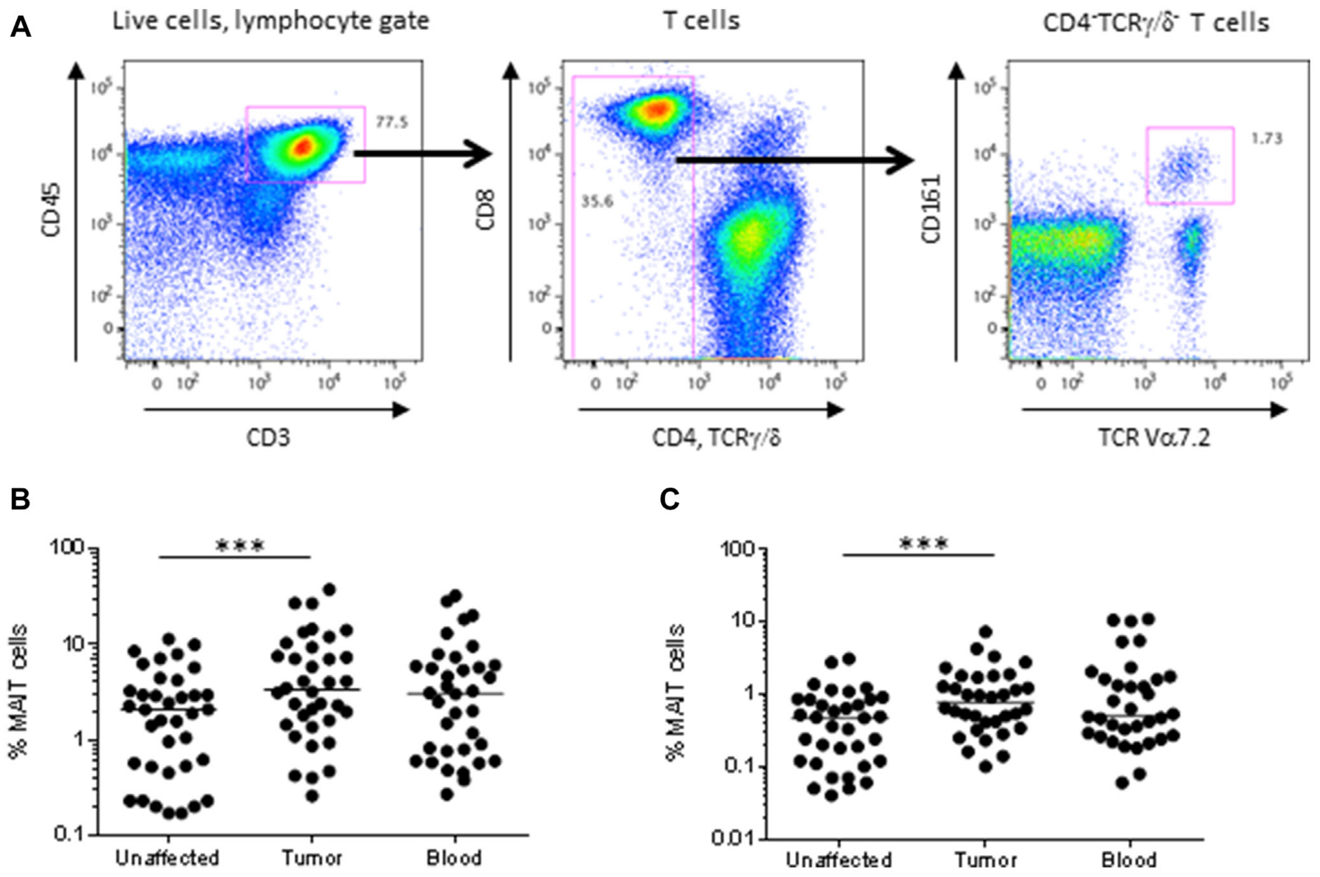


**Supplementary Figure 1: Gating strategy to identify MAIT cells.** (**A**) Dot plot shows one example of MAIT cell gating using a single cell suspension from a tumor. Live cells from a lymphocyte gate were further gated as $CD45^+CD3^+$T cells, and then $CD8^+$ and double negative T cells were used to determine the frequency of $Va7.2^+CD161^{high}$ MAIT cells. (**B**) Frequencies of MAIT cells among $CD8^+$ and double negative T cells and (**C**) among all $CD3^+$T cells isolated from unaffected colon, tumor tissue and peripheral blood. Symbols represent individual values and the line the median $^{***}P < 0.001$, $n = 35$.

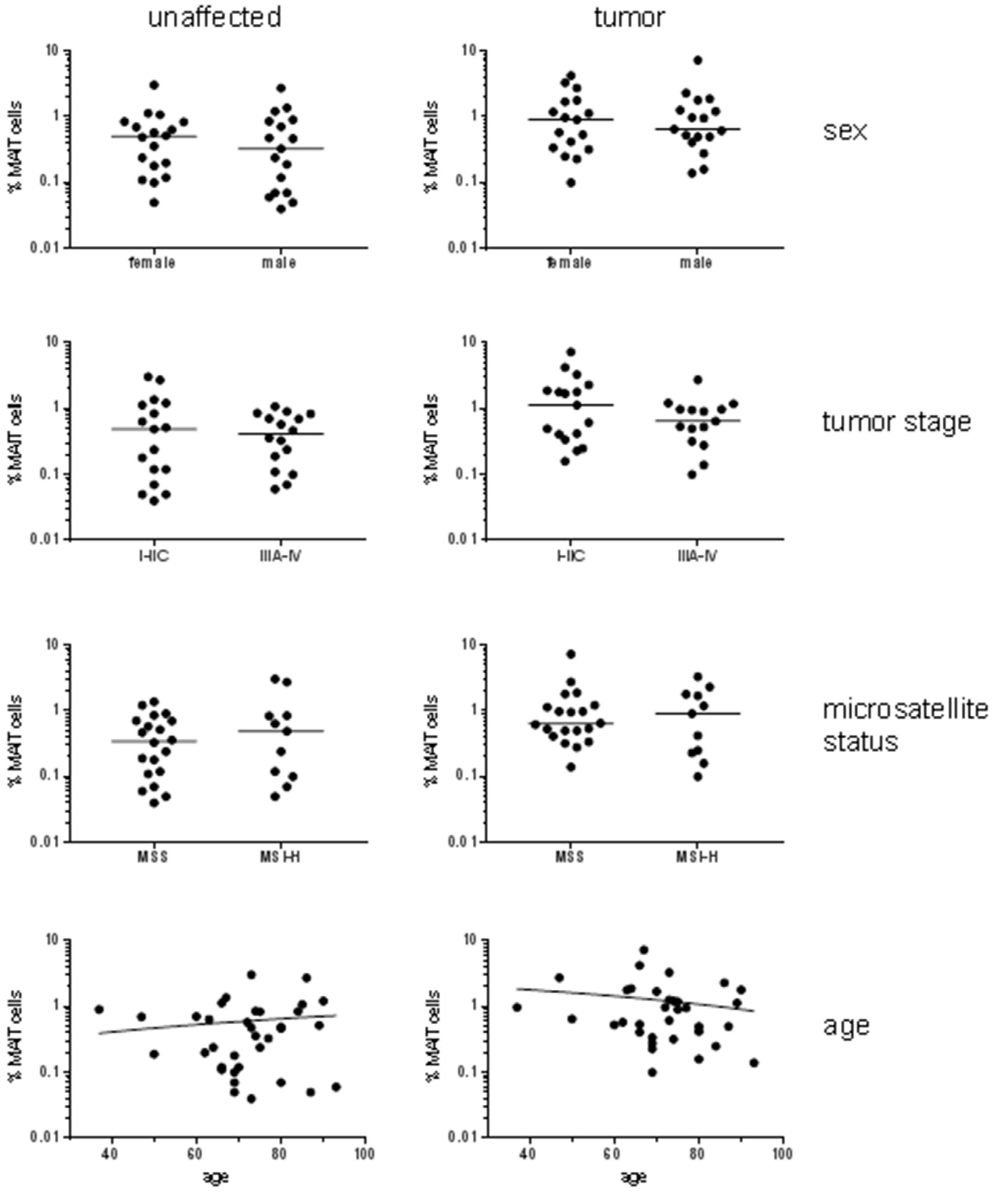


**Supplementary Figure 2: MAIT cell frequencies in relation to sex, tumor stage, microsatellite instability and age.** Single cell suspensions were prepared from unaffected colon tissue and tumors from the same patients, and the frequencies of MAIT cells in relation to all $CD3^+$ cells determined by flow cytometry. The other variables were retrieved from the pathology report *n* = 35.

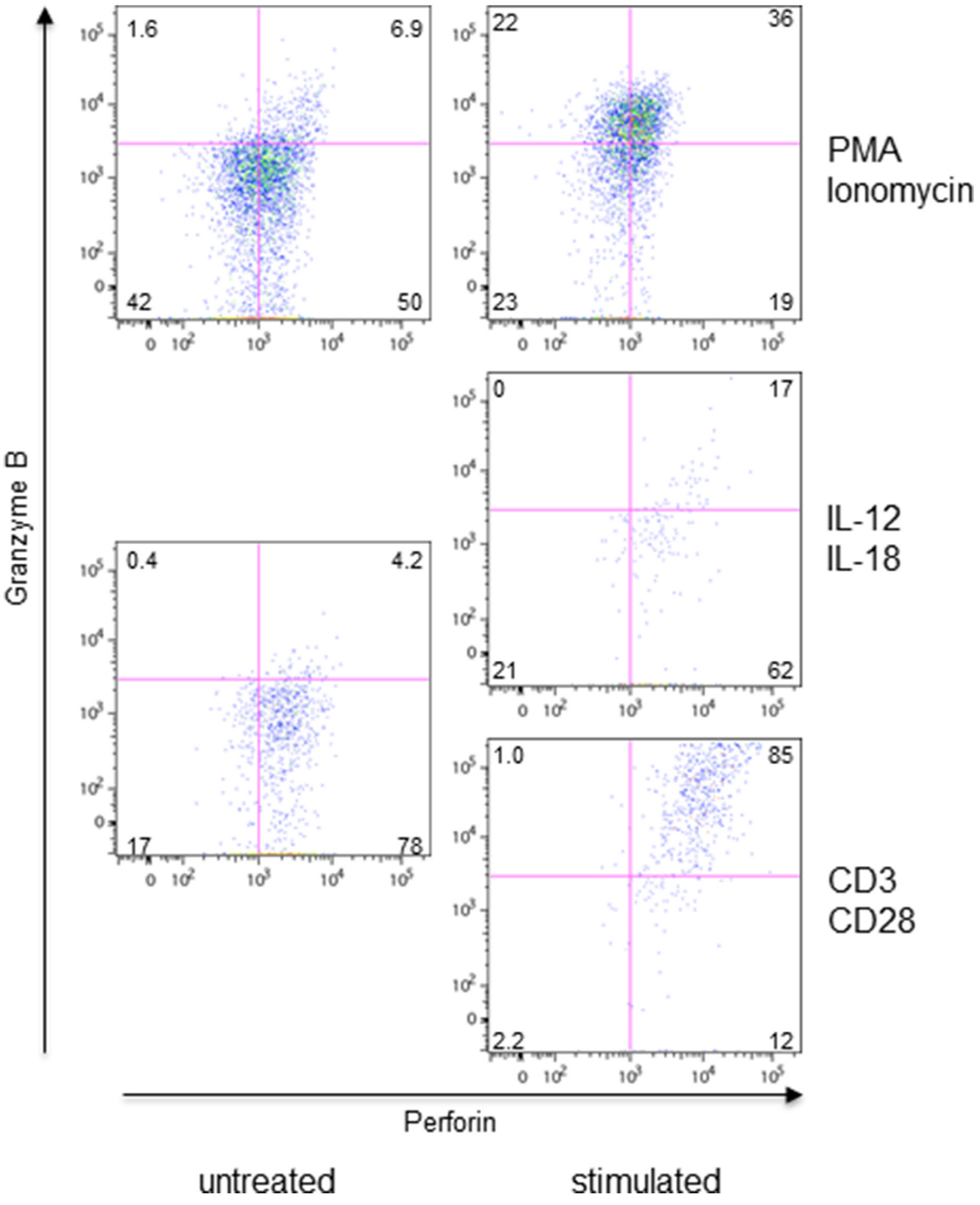


**Supplementary Figure 3: Representative FACS plots showing Granzyme B and Perforin staining in MAIT cells from colon tumors.** Single cell suspensions were isolated from colon tumors, and stimulated with PMA and Ionomycin, IL-12 and IL-18, or anti-CD3 and anti-CD28. MAIT cells were gated as in supplementary Figure 1, and expression of GrB and Perforin was examined by flow cytometry in untreated and stimulated cells.

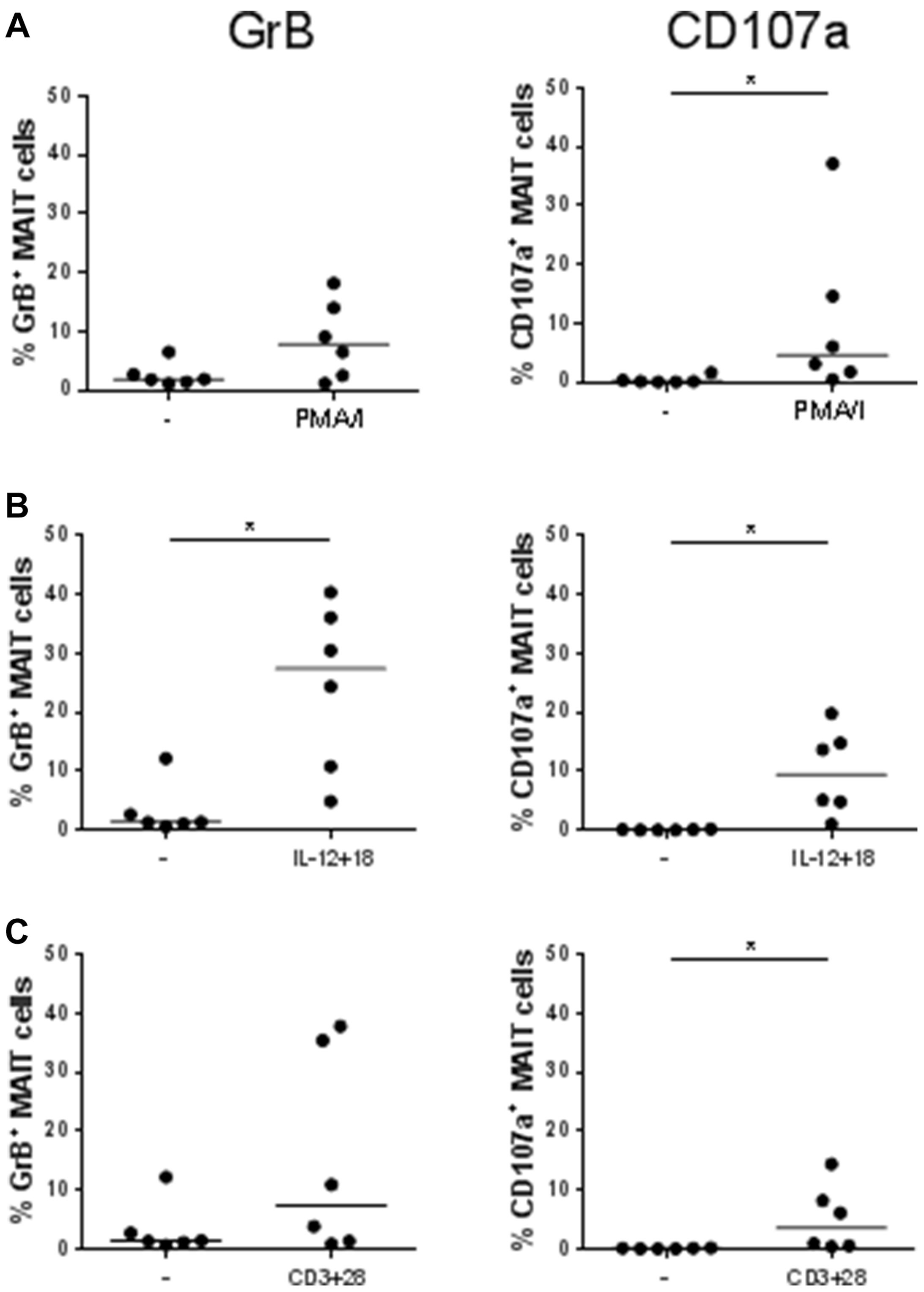


**Supplementary Figure 4: Frequencies of GrB+ and CD107a+ circulating MAIT cells from healthy volunteers.** Single cell suspensions were isolated from peripheral blood from healthy volunteers and stimulated with (**A**) PMA and Ionomycin, (**B**) IL-12 and IL-18, or (**C**) anti-CD3 and anti-CD28. Expression of GrB and CD107a was examined by flow cytometry. Symbols represent individual values and the line the median. $n = 6$ $^{*}p < 0.05$.

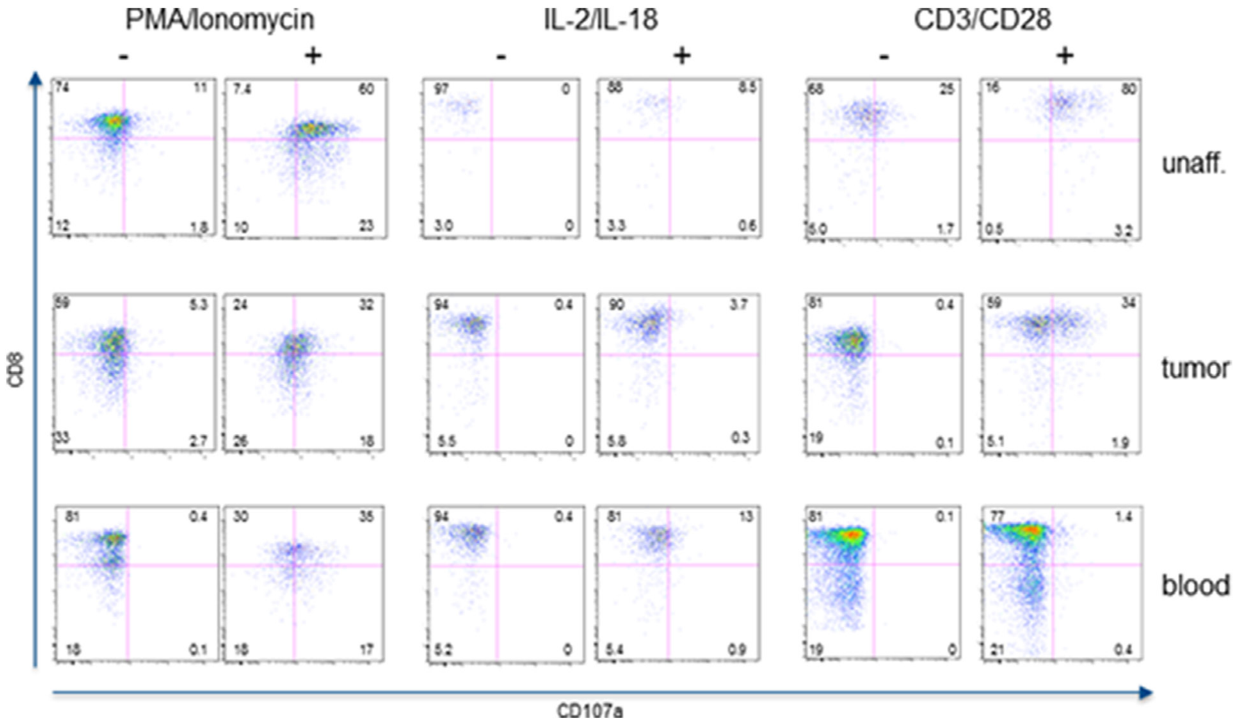


**Supplementary Figure 5: Degranulation of MAIT cells after stimulation.** Single cell suspensions were isolated from unaffected colon, colon tumors and peripheral blood, and stimulated with PMA and Ionomycin, IL-12 and IL-18, or anti-CD3 and anti-CD28. Expression if CD107a was examined by flow cytometry.

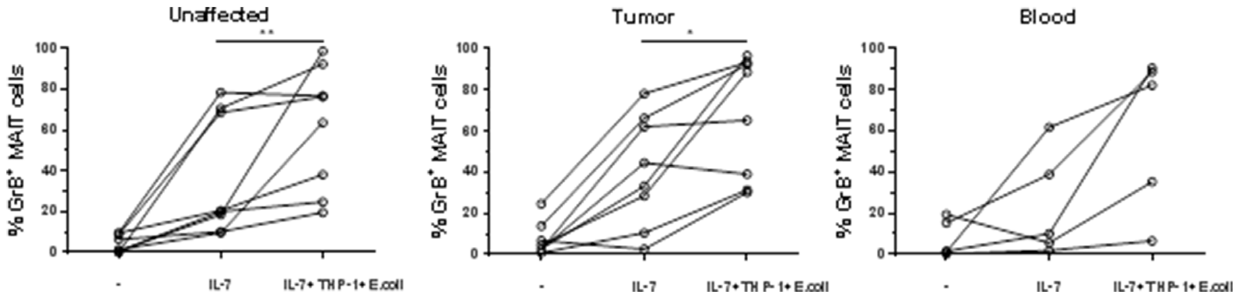


**Supplementary Figure 6: Frequencies of GrB$^{+}$ MAIT cells after antigen stimulation.** Single cell suspensions were isolated from unaffected colon, colon tumors and peripheral blood, and pre-incubated with IL-7 for 48 hours. They were then either left in IL-7 alone or co-cultured with THP-1 cells pre-incubated with fixed E. coli for 4 hours. Expression of GrB was examined by flow cytometry. Symbols represent individual values. $n$ = 5–8 $^{*}p < 0.05$, $^{**}p < 0.01$.

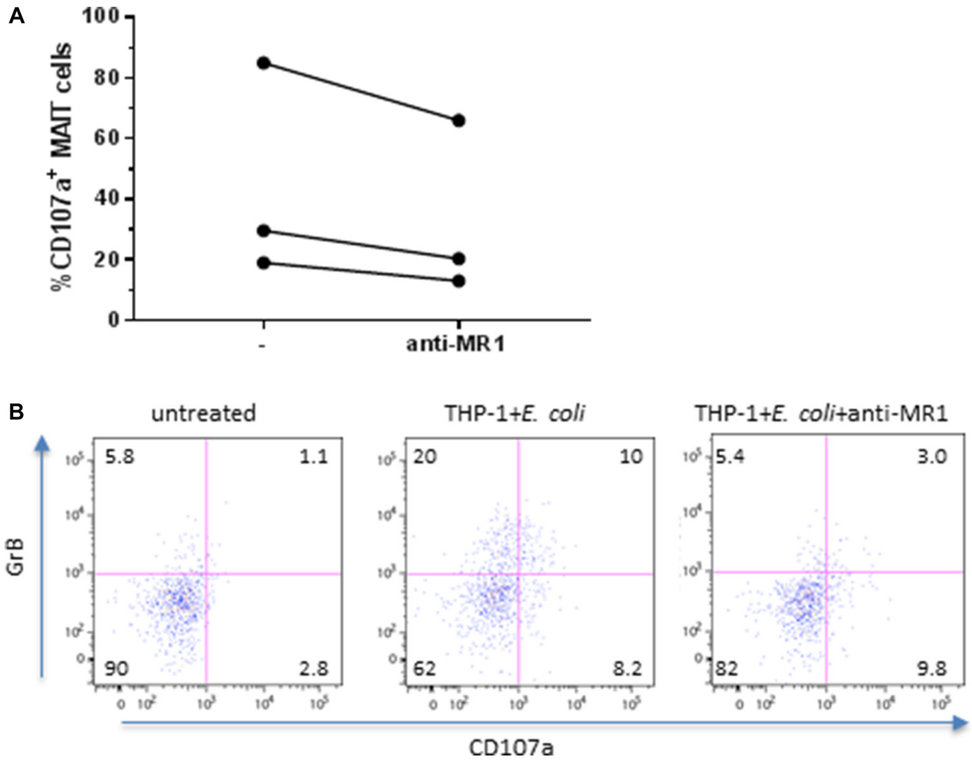


**Supplementary Figure 7: Degranulation of MAIT cells in the presence of antibodies to MR1.** Single cell suspensions were isolated from colon tumors and pre-incubated with IL-7 for 48 hours. They were then stimulated for 18 hours with THP-1 cells pre-incubated with fixed *E. coli* in the presence or absence of 10 mg/ml of monoclonal antibody to MR1. Expression of CD107a on MAIT cells was examined by flow cytometry. Symbols represent individual values from 3 different donors (**A**), and dot plots (**B**) show representative staining of MAIT cells isolated from a colon tumor and left unstimulated, or stimulated in the absence of presence of anti-MR1.

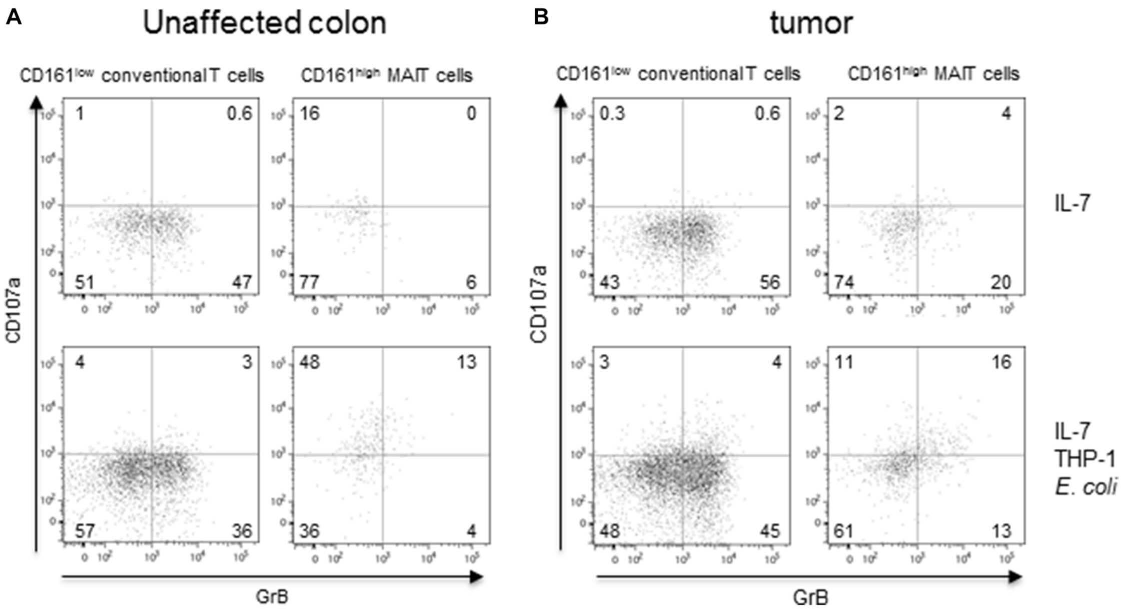


**Supplementary Figure 8: Degranulation by isolated colonic MAIT cells.** $CD8^+$ T cells were isolated by negative magnetic bead separation of single cell suspensions from unaffected colon and colon tumors, and incubated with IL-7 for 40 hours. $V\alpha7.2^+$ cells were then isolated by positive magnetic bead separation and co-cultured with THP-1 cells, which had previously been pre-incubated with fixed *E. coli* for four hours. Expression of GrB and CD107a on $Va7.2^+CD161^{high}$ and $Va7.2^+CD161^{low}$ T cells isolated from (**A**) unaffected colon and (**B**) tumor tissue was determined by flow cytometry in cells only pre-incubated with IL-7 or co-cultured with *E. coli*-loaded THP-1 cells. One representative experiment out of three is shown.

**Supplementary Table 1: Characteristics of the colon cancer patients included in the study**

| | | **females** | **males** |
|---|---|---|---|
| | n | 19 | 16 |
| | age | 47–92 | 37–93 |
| Tumor location | ascending | 10 | 11 |
| | transverse | 5 | 2 |
| | descending | 4 | 3 |
| TNM stage | I | 4 | 4 |
| | IIA | 5 | 4 |
| | IIB | 1 | - |
| | IIC | - | - |
| | IIIA | - | - |
| | IIIB | 5 | 5 |
| | IIIC | 4 | 3 |
| | IV | - | - |
| Microsatellite stability | MSS | 8 | 14 |
| | MSI-H | 9 | 2 |
| | unknown | 2 | - |
